# Sustained increase in depression and anxiety among psychiatrically healthy adolescents during late stage COVID-19 pandemic

**DOI:** 10.3389/fpsyt.2023.1137842

**Published:** 2023-03-17

**Authors:** Gabe Cochran, Zsofia P. Cohen, Martin P. Paulus, Aki Tsuchiyagaito, Namik Kirlic

**Affiliations:** ^1^Laureate Institute for Brain Research, Tulsa, OK, United States; ^2^Department of Psychology, Oklahoma State University, Stillwater, OK, United States; ^3^Oxley College of Health Sciences, The University of Tulsa, Tulsa, OK, United States; ^4^Research Center for Child Mental Development, Chiba University, Chiba, Japan

**Keywords:** depression, anxiety, adolescent, COVID-19, mental health, emotion regulation

## Abstract

**Background:**

Adolescents have experienced increases in anxiety, depression, and stress during the COVID-19 pandemic and may be at particular risk for suffering from long-term mental health consequences because of their unique developmental stage. This study aimed to determine if initial increases in depression and anxiety in a small sample of healthy adolescents after the onset of the COVID-19 pandemic were sustained at follow-up during a later stage of the pandemic.

**Methods:**

Fifteen healthy adolescents completed self-report measures at three timepoints (pre-pandemic [T1], early pandemic [T2], and later pandemic [T3]). The sustained effect of COVID-19 on depression and anxiety was examined using linear mixed-effect analyses. An exploratory analysis was conducted to investigate the relationship between difficulties in emotion regulation during COVID-19 at T2 and increases in depression and anxiety at T3.

**Results:**

The severity of depression and anxiety was significantly increased at T2 and sustained at T3 (depression: Hedges’ g _[T1 to T2]_ = 1.04, g _[T1 to T3]_ = 0.95; anxiety: g _[T1 to T2]_ = 0.79, g _[T1 to T3]_ = 0.80). This was accompanied by sustained reductions in positive affect, peer trust, and peer communication. Greater levels of difficulties in emotion regulation at T2 were related to greater symptoms of depression and anxiety at T3 (rho = 0.71 to 0.80).

**Conclusion:**

Increased symptoms of depression and anxiety were sustained at the later stage of the pandemic in healthy adolescents. Replication of these findings with a larger sample size would be required to draw firm conclusions.

## 1. Introduction

Emerging literature shows that the rapid spread of COVID-19 has profoundly affected mental health in the general population, with reports indicating higher levels of anxiety, depression, stress, and even increased cases of stereotyping and discrimination (1–3). These effects are also seen in adolescent populations (4–7). Because adolescence is a sensitive psychosocial developmental period during which symptoms of mental illness may begin to appear, children and adolescents may be at particular risk for suffering from poor mental health as a consequence of the COVID-19 pandemic (8). Furthermore, adolescents may be at increased risk for decline in mental health due to increased isolation, increased exposure to parents’ mental health issues, disrupted routines as a result of Safer-at-Home orders, loss of milestones (e.g., graduation, homecoming) and schools switching to remote learning (9–11). Studies have identified that females, adolescents with pre-existing mental health conditions, and adolescents with low family support were the most at risk for increased depression and anxiety during the COVID-19 pandemic (12–14). Moreover, there is some evidence of an increased risk for familial violence during the pandemic (15).

In our previous report (7), we examined mental health symptoms among psychiatrically healthy adolescents and adolescents with histories of early life stress prior to and after the onset of the COVID-19 pandemic. Contrary to our hypotheses, we found that depression and anxiety symptoms were increased in healthy adolescents, whereas adolescents with histories of early life stress demonstrated no significant changes in already elevated symptoms (7). We reasoned that adolescents exposed to early life stress might not show elevated depression and anxiety as a result of their pre-existing chronic stress exposure, or the pandemic may have resulted in potentially buffering changes for those high-risk adolescents (e.g., enhanced feelings of closeness with family members, reduction in social obligations, etc.) (16); however, the pandemic may have significantly affected mental health, especially for adolescents without any pre-existing mental health conditions. Although a growing number of studies show acute changes in adolescent depression and anxiety at the onset of the pandemic (17–19), those have not documented whether changes are later sustained. Moreover, prior research reported that one’s ability to regulate their emotion plays an important role in determining the impact of unexpected crises on youth and adolescents (20, 21). The ability to regulate one’s emotion (i.e., emotion regulation) includes multiple processes such as monitoring, evaluating, modulating, and managing emotional experiences to achieve one’s goal (22, 23). Adolescent’s ability to regulate their emotions can be influenced by their previous interactions with the social environment (e.g., attachment, socialization processes, peer interactions, etc.) as well as their developmental stage (24, 25). Thus, individual differences in emotion regulation may be one of the important variables influencing adolescents’ mental health as a consequence of the COVID-19 pandemic.

This study focused on psychiatrically healthy adolescents and sought to evaluate adolescent depression and anxiety at three separate time points: pre-COVID-19 pandemic (August–December 2019; T1), after the onset of the COVID-19 pandemic (June 2020; T2), and at a later stage of the COVID-19 pandemic (March 2021; T3). For a detailed description of the three stages of the COVID-19 pandemic, as well as cases of COVID-19 and COVID-19 restrictions, please refer to Methods section 2.3. We hypothesized that adolescent depression and anxiety would be increased after the onset of the COVID-19 pandemic (T2) and sustained at the later stage of the COVID-19 pandemic (T3; primary outcomes). We also hypothesized that adolescent peer relationships, as well as mood state (i.e., positive and negative mood states), would be worsened after the onset of the COVID-19 pandemic (T2) and sustained at the later stage of the COVID-19 pandemic (T3; secondary outcomes). Finally, we explored whether increased levels of depression and anxiety at the later stage of the COVID-19 pandemic would be associated with peer relationships and difficulties in emotion regulation, or recognizing and managing affect (26), during the early stage of the COVID-19 pandemic (exploratory outcomes).

## 2. Methods

### 2.1. Participants

Psychiatrically healthy adolescents were eligible for the present study as part of a greater longitudinal investigation of (1) mood, anxiety, and stress disorders in adolescents [Neuroscience-Based Mental Health Assessment and Prediction for Adolescents (NeuroMAP-A)] and (2) the effects of real-time functional magnetic resonance imaging neurofeedback during mindfulness on adolescent resilience [Augmented Mindfulness Training for Resilience in Early Life (A-MindREaL)]. Both projects were funded by the National Institute for General Medical Sciences Centers of Biomedical Research Excellence (CoBRE) grant (1P20GM121312). Adolescents were recruited from the community using a school messaging platform (PeachJar), radio adverts, billboards, social network posts, news broadcasting, community presentations, word of mouth, and other miscellaneous methods. Eligibility was determined using a phone screen and remote and in-person visits with caregivers providing demographic information, medical and psychiatric history, pubertal status, and an MRI safety questionnaire. To be eligible for the present study, participants were required to be between 13 and 17 years of age at the time of study enrollment, fluent in English, have access to telephone, and have a consenting parent or guardian. Adolescents were excluded from participating for the following reasons: past or current psychiatric illness, endorsing two or more types of maltreatment on the Maltreatment and Abuse Chronology of Exposure Scale (MACE) (27), meeting the cutoff score on one or more of the five subscales on the Childhood Trauma Questionnaire (CTQ) (28), current use of medications affecting blood flow to the brain or brain function (i.e., methylphenidate, antipsychotics, acne medications), history of neurological disorders, magnetic resonance imaging contraindications, unwillingness or inability to complete major aspects of the study protocol, claustrophobia, non-correctable vision, hearing, or sensorimotor impairments, weight below 100 lbs.

### 2.2. Procedures

During a remote visit, a trained research assistant explained the purpose of the study and study procedures to eligible adolescents and caregivers, who then provided informed assent and consent, respectively, and electrically signed those documents. Procedures were approved by the Western Institutional Review Board. Fifteen adolescents completed online surveys pre-COVID-19 pandemic (T1) and after the onset of the COVID-19 pandemic (T2), and 13 adolescents completed online surveys at a later stage of the COVID-19 pandemic (T3). Of those 15 adolescents, zero reported that they and their household members had tested positive for or been diagnosed with COVID-19 at T2, and one reported that a non-household member had been diagnosed with COVID-19 at T2.

#### 2.2.1. Primary outcomes

The severity of depression was measured using the pediatric Patient-Reported Outcomes Measurement Information System (PROMIS)-Depression (29–32), and the severity of anxiety was measured by the pediatric PROMIS-Anxiety. The pediatric version of the PROMIS-Depression and -Anxiety were clinically validated through confirmatory factor analysis (confirming two factors: depression and anxiety) and item response theory (32). A T-score of 50 is the average for the United States general youth population with a standard deviation (SD) of 10. A higher T-score represents greater depression or anxiety severity, with a T-score between 55 and 60 indicating mild, 60–70 indicating moderate, and over 70 indicating severe depression or anxiety (33). In the present sample, the PROMIS had high internal consistency within each subscale (depression: ⍺ = 0.91, anxiety: ⍺ = 0.92).

#### 2.2.2. Secondary outcomes

The child version of the Positive and Negative Affect Schedule (PANAS-C) (34) was used to measure positive and negative affect. Positive affect refers to the propensity to experience positive emotions and expressions such as joy, cheerfulness, or calmness, and negative affect refers to the propensity to experience negative emotions such as anger, fear, or sadness. The PANAS-C demonstrated good convergent and discriminant validity (34). In the present sample, the PANAS-C had high internal consistency within each subscale (positive affect: ⍺ = 0.94, negative affect: ⍺ = 0.90).

Peer relationships were assessed using the peer version of the Inventory of Parent and Peer Attachment-Revised (IPPA-R) for Children (35). Peer trust refers to the adolescents’ trust that their peers respect and understand their needs and goals; peer communication refers to adolescents’ perceptions that peers are responsive to their emotional states, as well as assessing the amount and quality of verbal communication with them; and peer alienation refers to adolescents’ feelings of isolation, anger, and detachment experienced in relationships with peers (36). The IPPA-R demonstrated adequate to good internal consistency and adequate convergent validity (35). We used peer trust and peer communication subscales since the peer alienation subscale revealed poor internal consistency in the current sample (⍺ = 0.49). Peer trust and peer communication subscales showed high internal consistency (peer trust: ⍺ = 0.96, peer communication: ⍺ = 0.90).

#### 2.2.3. Exploratory outcomes

To assess difficulties in emotion regulation during the COVID-19 pandemic, we used the Difficulties in Emotion Regulation Scale (DERS) (37). The DERS demonstrated high internal consistency, good test–retest reliability, and adequate construct and predictive validity (37). The original scale was adapted for the current study by adding the word “during this pandemic” to each item (e.g., “During this pandemic, I am experiencing my emotions as overwhelming and out of control”). The DERS measures difficulties in emotion regulations in six domains: (1) nonacceptance of emotional responses reflects a tendency to have a non-accepting reaction to one’s own distress; (2) difficulty engaging in goal-directed behavior reflects difficulty in concentrating or accomplishing tasks when experiencing negative emotions; (3) impulse control difficulties refers to difficulty remaining in control of one’s behavior when experiencing negative emotions; (4) lack of emotional awareness reflects a lack of awareness or inattention to emotional responses; (5) limited access to emotion regulation strategies reflects one’s belief that there is little one can do to regulate oneself once upset; and (6) lack of emotional clarity reflects the extent to which an individual knows and is clear about one’s emotions. Higher scores of each subscale indicate more emotion regulation problems in each domain. In the present sample, the DERS had high overall internal consistency (⍺ = 0.96) as well as good internal consistency within each subscale (⍺ ≥ 0.86).

#### 2.2.4. Other COVID-19 related assessments

We also administered the COVID-19 Adolescent Symptom and Psychological Experience Questionnaire (CASPE) (38) and the Coronavirus Health Impact Survey (CRISIS) (39). The summary statements of those scales are available in the Supplementary Table S1.

Primary and secondary outcomes were collected at all three time points, while exploratory outcomes were collected only at T2.

### 2.3. COVID-19 context

The present study was conducted in Tulsa, Oklahoma. The first case of COVID-19 in Oklahoma was announced on March 7^th^, 2020. The WHO categorized the disease as a worldwide pandemic on March 11^th^ of the same year. Public schools began announcing closures on March 20^th^, and the city of Tulsa issued “Safer-at-Home” orders on the 28^th^. These included the closure of non-essential businesses and encouraging solely essential trips from the household. These orders applied to all Tulsa residents. Cases of COVID-19 and attributed deaths continued to rise, and by the conclusion of data collection for the second timepoint in June 2020, the total number of infections was 9,354 and deaths was 366. Notably, all COVID-19 restrictions were lifted by June 1^st^. Surveys for the second timepoint of the present study were administered on May 22, 2020, and completed by June 18^th^, 2020. Between baseline assessments and timepoint 2, participants had experienced the pandemic onset, a shelter-in-place order, increasing local cases, and eventually reopening. The first COVID-19 vaccine was approved in December of 2020 and was slowly made available to the community in the following months. Follow-up surveys (T3) were administered and completed in March of 2021. By this point, local schools had reopened, and many adopted a hybrid model, transitioning to virtual learning in the event of rising infections in the school. Near the conclusion of data collection in February 2021, there had been 69,112 confirmed cases of COVID-19 in Tulsa county and 661 deaths (40).

### 2.4. Statistical analysis

Statistical analyses were performed using R version 4.1.0. Linear mixed-effect analyses (LME: *lme4* package (41)) were conducted to examine the change in primary and secondary outcomes. The LME model included a fixed effect of time (T1, T2, and T3) and a subject as a random effect. Post-hoc comparisons were conducted with Tukey’s method for the primary outcomes. Next, Spearman’s partial correlations were calculated to investigate the relationship between symptoms of depression and anxiety at T3 and peer trust, peer communication, and difficulties in emotion regulation at T2, controlling for symptoms of depression and anxiety at T1. *p*-values for the correlation analysis were corrected with the false discovery rate (FDR).

## 3. Results

### 3.1. Demographics and COVID-19 pandemic-related experiences

**Figure 1:**
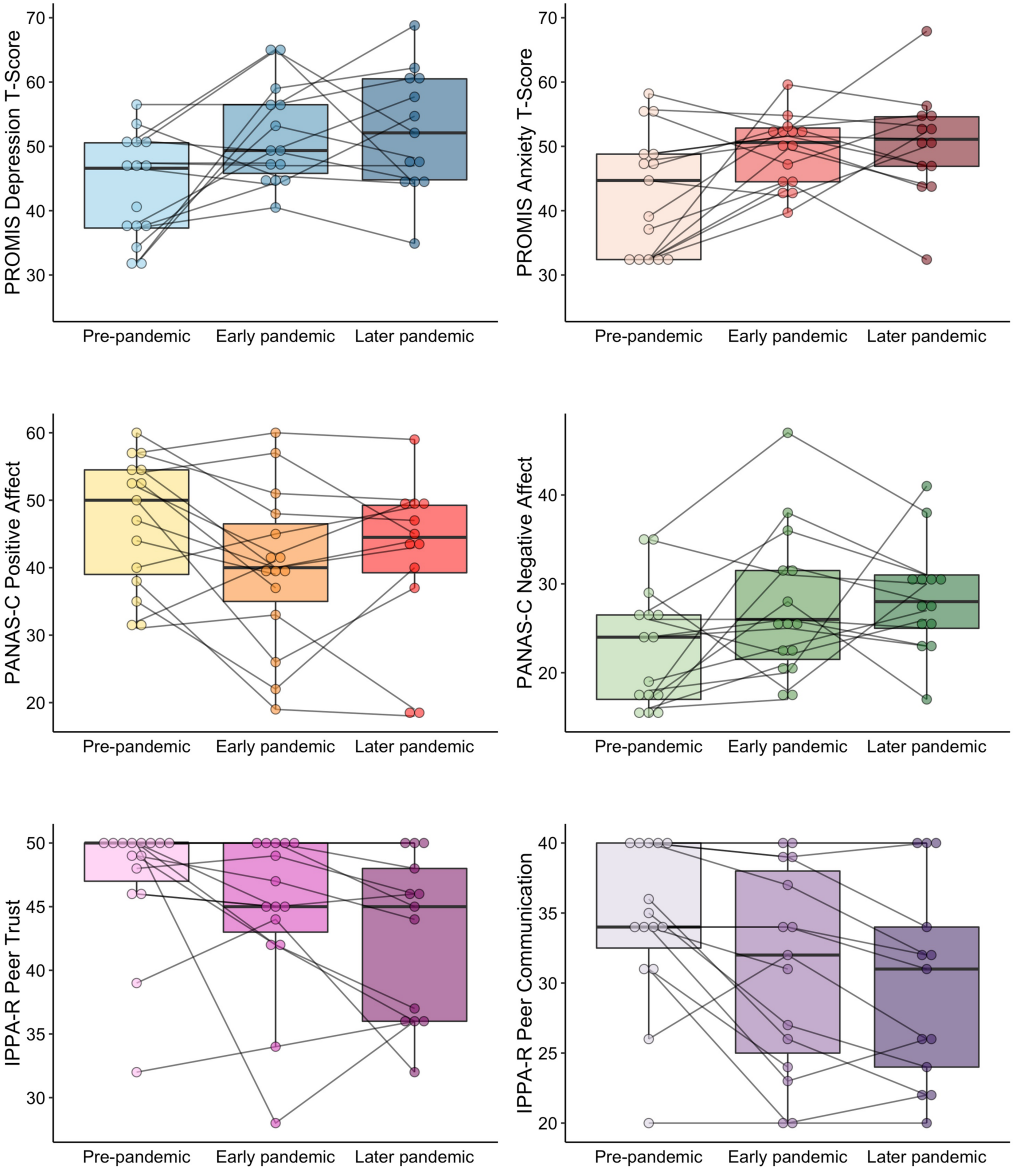
Individual and box plots of the primary and secondary outcomes across three time points.

Demographics of participants and the summary of the COVID-19 pandemic-related experiences are summarized in Table 1 and Supplementary Table S1, respectively, and changes in outcomes are summarized in Table 2. Individual and box plots of the primary and secondary outcomes are illustrated in Figure 1.

**Table 1:** Demographics.

		Mean	SD
Age at pre-COVID-19 pandemic		14.53	1.30
Parent Income/Gross Family Income		102,066.67	80,293.63
Grade in school		8.67	1.23
		*n*	%
Gender	Male	9	60.00
	Female	6	40.00
	Non-binary	0	0.00
Ethnicity	Hispanic	2	13.30
	Non-Hispanic	13	86.60
Race	White	12	80.00
	Black/African American	2	13.33
	American Indian/Alaska Native	3	20.00
	Asian Indian	1	6.67

**Table 2:** Outcomes at pre-pandemic (T1), early pandemic (T2), and later pandemic (T3).

		T1	T2	T3
		Mean (SD)	Mean (SD)	Mean (SD)
**Primary outcomes**				
PROMIS	Depression	43.65 (8.20)	52.95 (9.13)	52.31 (9.48)
	Anxiety	42.95 (9.49)	49.20 (5.46)	50.31 (8.30)
**Secondary outcomes**				
PANAS-C	Positive affect	47.00 (9.70)	40.00 (11.72)	41.75 (12.20)
	Negative affect	22.93 (6.73)	27.33 (8.26)	28.46 (6.31)
IPPA-R	Peer trust	47.27 (5.15)	44.73 (6.40)	42.77 (6.44)
	Peer communication	34.33 (5.74)	31.07 (7.27)	29.92 (7.19)
**Exploratory outcomes**				
DERS	Total score	-	66.67 (22.97)	-
	Nonacceptance of emotional responses	-	8.20 (4.52)	-
	Difficulty engaging in goal-directed behavior	-	11.27 (4.86)	-
	Impulse control difficulties	-	9.07 (3.97)	-
	Lack of emotional awareness	-	14.67 (5.70)	-
	Limited access to emotion regulation strategies	-	13.93 (5.34)	-
	Lack of emotional clarity	-	9.53 (4.49)	-

Means and standard deviations (SD) of outcome measures at pre-pandemic (T1), early pandemic (T2), and later pandemic (T3). PROMIS, pediatric patient-reported outcomes measurement information system; PANAS-C, positive and negative affect schedule-child version; IPPA-R, inventory of parent and peer attachment-revised for children; DERS, difficulties in emotion regulation questionnaire; DERS, Difficulties in emotion regulation questionnaire. PROMIS scores are reported as T-scores.

### 3.2. Changes in primary outcomes

#### 3.2.1. Depression

There was a significant time effect on PROMIS depression T-scores [*F*(2, 27.16) = 5.28, *p* = 0.01, R^2^ = 0.20]. A *post-hoc* analysis revealed increases in symptoms of depression from T1 to T2 (z = 2.95, *p* = 0.01, Hedges’ g = 1.04), which were maintained at T3 (T1 to T3: z = 2.62, *p* = 0.02, g = 0.95; T2 to T3: z = −0.22, *p* = 0.97, g = −0.07). Based on the T-score at T3, 38% of our sample were still experiencing mild levels of depression, while 40% showed mild levels of depression at T2.

#### 3.2.2. Anxiety

There was a significant time effect on PROMIS anxiety T-score [*F*(2, 26.37) = 5.30, *p* = 0.012, R^2^ = 0.14]. A *post-hoc* analysis revealed increases in symptoms of anxiety from T1 to T2 (z = 2.75, *p* = 0.02, g = 0.79), which were maintained at T3 (T1 to T3: z = 2.85, *p* = 0.01, g = 0.80; T2 to T3: z = 0.23, *p* = 0.97, g = 0.16). Based on the T-score at T3, 15% of our sample were still experiencing mild anxiety, while 6% showed mild levels of anxiety at T2.

### 3.3. Changes In secondary outcomes

#### 3.3.1. Positive affect

There was a significant time effect on PANAS positive affect [*F*(2, 25.66) = 4.86, *p* = 0.02, R^2^ = 0.08]. A *post-hoc* analysis revealed reductions in positive affect from T1 to T2 (z = −2.96, *p* = 0.01, g = −0.63), which were maintained at T3 (T1 to T3: z = −2.24, *p* = 0.06, g = −0.47; T2 to T3: z = 0.49, *p* = 0.88, g = 0.14).

#### 3.3.2. Negative affect

There was a trend-wise significant time effect on PANAS negative affect [*F*(2, 25.98) = 3.04, *p* = 0.06, R^2^ = 0.09]. A *post-hoc* analysis revealed a trend-wise increase in negative affect from T1 to T3 (z = 2.19, *p* = 0.07, g = 0.82), but not from T1 to T2 and from T2 to T3 (T1 to T2: z = 2.04, *p* = 0.10, g = 0.57; T2 to T3: z = 0.24, *p* = 0.97, g = 0.15).

#### 3.3.3. Peer trust

There was a significant time effect on IPPA-R peer trust [*F*(2, 26.65) = 4.31, *p* = 0.02, R^2^ = 0.09]. A *post-hoc* analysis revealed reductions in peer trust from T1 to T3 (z = −2.91, *p* = 0.01, g = −0.76), while there was no significant difference between T1 to T2 and T2 and T3 (T1 to T2: z = −1.74, *p* = 0.19, g = −0.42; T2 to T3: z = −1.25, *p* = 0.42, g = −0.30).

#### 3.3.4. Peer communication

There was a significant time effect on IPPA-R peer communication [*F*(2, 26.25) = 8.19, *p* = 0.002, R^2^ = 0.08]. A *post-hoc* analysis revealed reductions in peer trust from T1 to T2 (z = −2.81, *p* = 0.01, g = −0.49), which were maintained at T3 (T1 to T3: z = −3.90, *p* = 0.001, g = −0.66; T2 to T3: z = −1.23, *p* = 0.44, g = −0.15).

### 3.4. Correlations between primary outcomes and exploratory outcomes

Regardless of the baseline levels of depression and anxiety (T1), greater levels of difficulties in emotion regulation during the early stage of the COVID-19 pandemic (T2) were related to greater symptoms of depression and anxiety at the later stage of the COVID-19 pandemic (T3) (Table 3; rho = 0.71 to 0.80, *p*-FDR < 0.05). Especially, limited access to emotion regulation strategies at T2 was strongly correlated with both depression and anxiety severity at T3 (depression: rho = 0.78, anxiety: rho = 0.80). The relationship between peer trust, peer communication, difficulties in emotion regulation during the early pandemic (T2) and depression and anxiety at the same time point (T2) are summarized in the Supplementary Table S2.

**Table 3:** Spearman’s partial correlations between difficulties in emotion regulation at the early pandemic (T2) and depression/anxiety severity at the later pandemic (T3).

		Later COVID-19 pandemic (T3)					
		PROMIS Depression			PROMIS Anxiety		
Early COVID-19 pandemic (T2)		rho	*p_uncorrcted_*	*p_FDR_*	rho	*p_uncorrcted_*	*p_FDR_*
**IPPA-R**							
	Peer trust	0.28	0.38	0.38	0.26	0.42	0.54
	Peer communication	0.32	0.30	0.34	0.30	0.35	0.54
**DERS**							
	Total score	0.71	0.01*	0.03*	0.37	0.24	0.54
	Nonacceptance of emotional responses	0.34	0.28	0.34	0.41	0.18	0.54
	Difficulty engaging in goal-directed behavior	0.38	0.22	0.33	0.23	0.48	0.54
	Impulse control difficulties	0.59	0.04*	0.10	0.26	0.41	0.54
	Lack of emotional awareness	0.50	0.10	0.17	0.20	0.54	0.54
	Limited access to emotion regulation strategies	0.78	<0.01**	0.03*	0.80	<0.01**	0.01*
	Lack of emotional clarity	0.73	0.01*	0.03*	0.44	0.15	0.54

Spearman’s partial correlation (rho) after controlling for PROMIS Depression or Anxiety at pre-COVID-19 pandemic (T1). PROMIS, pediatric patient-reported outcomes measurement information system; DERS, difficulties in emotion regulation questionnaire. pFDR, *p*-values are adjusted with the false discovery rate (FDR) correction.

* *p* < 0.05;

** *p* < 0.01.

## 4. Discussion

Findings from our study are consistent with previous studies reporting sustained increases in anxiety and depression in adolescents as a result of the COVID-19 pandemic (42). Research on the longer-term trajectories of mental health symptoms during the first and second years of the COVID-19 pandemic is scarce. Few studies have shown that depression and anxiety symptoms have remained stable at later stages of the pandemic without obvious signs of improvement (43, 44). One study reported that healthy adults experienced sustained increases in levels of depressive and worry symptoms a year after the onset of the pandemic, but adults with psychiatric symptoms did not (43). Our results add preliminary evidence that psychiatrically healthy adolescents may be experiencing heightened depression and anxiety even at a later stage of the pandemic (33). This was accompanied by elevated negative mood as well as lower positive mood. Also, adolescents’ perception of lower levels of peer trust and communication was sustained at the later pandemic.

Although prior studies showed that peer support may help adolescents attain better well-being and address mental health needs (45, 46), in our exploratory analysis, peer relationship at the arly pandemic was not associated with depression and anxiety later in the pandemic. Rather, our results highlight the role of trait emotion regulation. Specifically, adolescents who reported that they had less emotion regulation strategies early in the pandemic were also more likely to later report greater levels of depression and anxiety. Those associations were observed controlling for the baseline levels of depression and anxiety before the onset of the pandemic. A recent study reported that individual differences in emotion regulation difficulties before the pandemic predicted greater COVID-19 acute stress in young adults (47). Our results also supported the notion that emotion regulation may be one of the vulnerabilities increasing risk for developing depression and/or anxiety following an unexpected and uncertain stressful event, such as the COVID-19 pandemic. Adolescents may benefit from having a flexible and diverse repertoire of emotion regulation strategies under stressful circumstances (47, 48).

It is important to note that emotion regulation skills develop substantially across adolescence. Studies of typically developing individuals indicate that adolescents with limited efficacy of emotion regulation strategies in early adolescence, shift to increased use of adaptive emotion regulations (e.g., emotional clarity, reappraisal, and social support seeking, etc.) and decreased use of maladaptive emotion regulations (e.g., suppression, rumination, and avoidance, etc.) with age (49–52). Therefore, enhancing the development of emotion regulation strategies by teaching adaptive emotion regulation skills such as emotional awareness, acceptance, mindfulness, or managing negative feelings with cognitive reappraisal can help adolescents better regulate their emotions and thoughts during crisis. Additionally, there are individual differences such as sex, pubertal stage, and cognitive ability that need to be considered in understanding how emotion regulation skills develop. Future research may be interested in building individualized materials to teach adaptive emotion regulation skills, depending on those factors since they have been mentioned as having an effect on the connection between emotion regulation and psychopathology (52–56).

## 5. Limitations

The availability of pre-pandemic data and the longitudinal design of the present study provides the opportunity for interesting analyses; however, we are limited by the small sample size (*n* = 15). Especially, the exploratory correlational analysis needs to be interpreted with caution since the analyses are correlational, and the results could have been influenced by other variables. Results from the present study should be replicated with larger sample sizes. Moreover, the timing of the later stage of COVID-19 was not strictly defined but rather based on the COVID restrictions and regulations in the Tulsa county area, while several studies defined the second year of the pandemic or February to March 2021 as the later pandemic (43, 44).

## 6. Conclusion

The present study indicates that psychiatrically healthy adolescents may be experiencing a sustained increase in depression and anxiety symptoms at later stages of the COVID-19 pandemic. We further provide preliminary results of the relationship between emotion regulation and the later course of depression and anxiety. These findings indicate a potential need to offer flexible and diverse repertoire of emotion regulation strategies for adolescents in the face of unexpected stressful events.

## Data availability statement

The raw data supporting the conclusions of this article will be made available by the authors, without undue reservation.

## Ethics statement

The studies involving human participants were reviewed and approved by WCG IRB. Written informed consent to participate in this study was provided by the participants’ legal guardian/next of kin.

## Author contributions

GC, ZC, MP, AT, and NK: conceptualization and methodology. GC, AT, and NK: formal analysis. GC and AT: writing – original draft. ZC, MP, and NK: writing – review and editing. MP and NK: supervision and funding acquisition. All authors contributed to the article and approved the submitted version.

## Funding

This work has been supported in part by the Laureate Institute for Brain Research, and the National Institute of General Medical Sciences Center Grant Award Number (1P20GM121312). The content is solely the responsibility of the authors and does not necessarily represent the official views of the National Institutes of Health.

## Conflict of interest

MP is an advisor to Spring Care, Inc., a behavioral health startup, he has received royalties for an article about methamphetamine in UpToDate.

The remaining authors declare that the research was conducted in the absence of any commercial or financial relationships that could be construed as a potential conflict of interest.

## Publisher’s note

All claims expressed in this article are solely those of the authors and do not necessarily represent those of their affiliated organizations, or those of the publisher, the editors and the reviewers. Any product that may be evaluated in this article, or claim that may be made by its manufacturer, is not guaranteed or endorsed by the publisher.
