# Supplementary material for: Sustained increase in depression and anxiety among psychiatrically healthy adolescents during late stage COVID-19 pandemic
Source: Front Psychiatry. 2023 Mar 17;14:1137842. doi: 10.3389/fpsyt.2023.1137842 (PMC10063786; doi:10.3389/fpsyt.2023.1137842)
Supplement: Supplementary file 1 [file Data_Sheet_1.PDF]

Supplementary Tables

Table S1. COVID-19 Pandemic-Related Experiences

Measure	Characteristic	n=15	
		n	%
Coronavirus Health Impact Survey (CRISIS)			
	COVID Symptoms		
	Cough	0	0%
	Fatigue	2	13%
	Fever	1	7%
	Loss of Taste or Smell	0	0%
	None	13	87%
	Shortness of Breath	0	0%
	Sore Throat	0	0%
	Family Diagnosis		
	Yes, Household member	0	0%
	Yes, Non-household member	1	7%
	No	14	93%
	Suspected Diagnosis		
	Positive Test	0	0%
	Possible Symptoms, No Diagnosis	1	7%
	Medical Diagnosis, No Test	0	0%
	Not Suspected	15	100%
	Outcomes due to COVID-19 for family members		
	Fallen Ill Physically	0	0%
	Hospitalized	1	7%
	Loss of Job	2	13%
	Passed Away	0	0%
	Reduced Ability to Earn Money	5	33%
	Self-Quarantine with Symptoms	2	13%
	Self-Quarantine without Symptoms	3	20%
	None	8	53%
	Exposure to someone with COVID-19		
	Positive Test	0	0%
	Possible Symptoms, No Diagnosis	1	7%
	Medical Diagnosis, No Test	0	0%
	No	14	93%
	Household Essential Workers		

Yes	9	60%
No	6	40%
Situations Applied to your Household		
Local government encouraging people to stay home	14	93%
Parent is a medical professional in quarantine	2	13%
Stay-in-order by local government	10	67%
Voluntary quarantine due to confirmed case	3	20%
Voluntary quarantine due to fear of exposure	8	53%
Activities your Family Stopped		
Contact with Family Inside the Home	1	7%
Contact with Family Outside the Home	8	53%
Contact with Friends Indoors	7	47%
Contact with Friends Outdoors	2	13%
Family Travel	9	60%
Family Activities in Outdoor Spaces	5	33%
Family Activities in Public Spaces	10	67%
Going to Restaurants or Stores	12	80%
Indoor Exercise	7	47%
In-Person Community Events	9	60%
In-Person Religious Services	9	60%
How are you coping with the stress or anxiety related to the COVID-19 outbreak?		
Arts and Crafts	4	27%
Board Games or Cards	9	60%
Drinking Alcohol	0	0%
Eating Comfort Foods	9	60%
Eating Healthier	2	13%
Exercising	8	53%
Getting a Good Night's Sleep	11	73%
Helping Others	2	13%
Increased Self-Care Activities	4	27%
Listening to Music	13	87%
Meditation	2	13%
More Family Activities	8	53%
Not Skipping Prescribed Medications	0	0%

	Playing an Instrument	4	27%
	Playing Video Games	12	80%
	Prayer	4	27%
	Reading	7	47%
	Spending Time with Pets	10	67%
	Taking New Prescription Drugs	0	0%
	Taking Vitamins	2	13%
	Talking to Friends	11	73%
	Talking to Mental Health Professionals	2	13%
	Texting or Other Social Media	10	67%
	Using Marijuana	2	13%
	Using Other Recreational Drugs	0	0%
	Using Tobacco	1	7%
	Watching Movies	8	53%
	Writing	2	13%

	Perceived impact of COVID-19	Mean	SD
COVID-19 Adolescent Symptom and Psychological Experience Questionnaire (CASPE)			
	Cognitive Disruptions	27.53	7.39
	Emotional Effects	12.13	4.03
	Extent of Negative Emotions	29.80	8.05
	Extent of Positive Emotions	15.53	3.87
	Has the quality of the relationships between you and members of your family changed?	3.33	0.90
	Has the quality of your relationships with your friends changed?	3.20	0.68
	How often are you getting outside of your house for allowed shelter-in-place activities?	2.53	1.64
	How often have you complied with social distancing or shelter-in place restrictions?	3.80	1.21
	How stressful have the restrictions on leaving home been for you?	2.40	1.35
	How worried have you been that someone in your house or extended family will get sick?	2.07	1.28

Level of concern related to impact of COVID19 pandemic	34.80	12.46
Negative Impact on Life	2.93	1.33
Positive Impact on Life	2.53	1.13
Stress regarding disruption of existing plans	2.33	1.23
Stress regarding uncertainty about the future	2.13	1.13

Note. Items are taken from the CASPE and CRISIS measures. Percentages are rounded to the nearest whole number. For items relating to quantity or quality, larger numbers indicate greater frequency or higher quality on the respective domain, with the exception of outside time, in which case greater time spent outdoors is indicated by a smaller score.

Table S2. Spearman's partial correlations between difficulties in emotion regulation (T2) and depression/anxiety severity at the early pandemic (T2).

	Early COVID-19 pandemic (T2)					
	PROMIS Depression			PROMIS Anxiety		
Early COVID-19 pandemic (T2)	rho	$p_{uncorrcted}$	p_{FDR}	rho	$p_{uncorrcted}$	p_{FDR}
IPPA-R						
Peer trust	0.16	0.59	0.59	0.25	0.38	0.49
Peer communication	0.36	0.21	0.24	0.25	0.38	0.49
DERS						
Total score	0.65	0.01*	0.02*	0.42	0.14	0.31
Nonacceptance of emotional responses	0.89	<0.001**	<0.001**	0.66	0.01*	0.10
Difficulty engaging in goal-directed behaviour	0.39	0.17	0.21	0.12	0.67	0.68
Impulse control difficulties	0.74	0.002**	0.01*	0.53	0.05	0.15
Lack of emotional awareness	0.40	0.16	0.21	0.12	0.68	0.68
Limited access to emotion regulation strategies	0.45	0.11	0.19	0.36	0.20	0.36
Lack of emotional clarity	0.69	0.01*	0.02*	0.54	0.04*	0.15

Spearman's partial correlation (rho) after controlling for PROMIS Depression or Anxiety at pre-COVID-19 pandemic (T1). PROMIS: pediatric Patient-Reported Outcomes Measurement Information System; DERS: Difficulties in Emotion Regulation Questionnaire. *$p < .05$, **$p < 0.01$. p_{FDR}: p-values are adjusted with the false discovery rate (FDR) correction.
